# Investigating epistemic emotions experienced while reading refutation texts through a fine-grained measure of emotion

**DOI:** 10.1038/s41539-025-00324-3

**Published:** 2025-05-15

**Authors:** Yi-Lun Jheng, Leen Catrysse, Sander Van de Cruys, Panayiota Kendeou, Karolien Poels, David Gijbels

**Affiliations:** 1 Department of Training and Educational Sciences, University of Antwerp, Antwerp, Belgium; 2 Department of Communication Studies, University of Antwerp, Antwerp, Belgium; 3 Department of Online Learning and Instruction, Faculty of Educational Sciences, Open Universiteit, Heerlen, the Netherlands; 4 Antwerp Social Lab, University of Antwerp, Antwerp, Belgium; 5 Department of Educational Psychology, University of Minnesota, Twin Cities, Minneapolis, MN USA

**Keywords:** Education, Communication, Psychology

## Abstract

The current study addressed the often-overlooked role of epistemic emotions in refuting misinformation by replicating and expanding on the work of Trevors and Kendeou (2020). It broadened the participant pool beyond well-educated college students and introduced a novel dynamic measure, “*DynamicEmo*”, to capture epistemic emotions experienced while reading refutation texts in a more fine-grained way. Results reaffirmed that positive, negative, and standard refutation texts (vs. non-refutation texts) effectively enhanced knowledge revision. Analysis using *DynamicEmo* revealed that paragraphs presenting inconsistent information (misinformation+correction) in refutation texts elicited activating (curiosity and confusion) or suppressed deactivating epistemic emotions (boredom). Notably, in-the-moment negative epistemic emotions, triggered by critical correct-outcome sentences, were negatively predictive of knowledge revision, highlighting the significance of emotions experienced during critical parts of refutation text reading. This study demonstrated the key role of epistemic emotions in knowledge revision, while offering more granular insights through dynamic emotion measurement compared to traditional post-hoc self-reports.

## Introduction

The spread of misinformation is a pressing issue in our contemporary society^1^. Misinformation is defined as any information that turns out to be false, whether it is spread accidentally or intentionally^2^. For instance, individuals may receive misleading information about vaccine safety or efficacy via text messages on various digital platforms forwarded to them by friends or family^3^. Misconceptions can be the result of misinformation; when people encounter and believe misinformation, they form misconceptions about a subject. A misconception is a belief or understanding that is incorrect. For example, videos containing misinformation circulating on social media can perpetuate misconceptions about vaccine safety, particularly among unvaccinated people^4^. Therefore, developing effective educational messages or techniques aimed at reducing misconceptions is of paramount importance. Refutational messages have been proven effective in debunking misconceptions as shown by recent reviews and meta-analyses^5–8^. In refutation texts, incorrect statements or misconceptions are directly refuted and correct information is provided accompanied by detailed explanations^9^. While research has been mostly focused on the underlying cognitive processes that make refutation messages effective^9–11^, we should acknowledge both cognitive factors (e.g., intuitive thinking) and socio-affective factors (e.g., emotion, worldview) as the drivers of false beliefs. Both likely have an impact on the effectiveness of refutation messages^5^.

Emotions are key to learning and information processing^12,13^. Epistemic emotions play a significant role in the effectiveness of refutation texts, especially when it comes to controversial or unsettled socio-scientific issues (e.g., immigration, vaccination issues)^14^. However, in a review of the literature on refutation texts, it was shown that only a small portion (13%) of studies specifically focused on the relation between emotions and learning from refutation texts^7^. Moreover, existing studies on emotions and refutation texts often rely on surveys administered after participants have read the texts^14–16^, failing to grasp in-the-moment emotions. Additionally, further exploration is needed to understand how changes in epistemic emotions during the reading of refutation texts influence knowledge acquisition outcomes, such as knowledge revision. For example, epistemic emotions such as curiosity, confusion, and frustration may arise when confronted with corrective information. Individuals may experience increased attention to resolve the cognitive dissonance caused by the refutation and thus result in deeper processing of the correct information. However, as the text unfolds and more explanations are provided, if the individuals experience negative emotions, such as threatened by the refutation explanations, they may be more inclined to reject or dismiss correct information and explanations, thereby hindering knowledge acquisition. To address this question and better understand in-the-moment emotions, we developed a novel “*DynamicEmo* measure” to capture the dynamic nature of emotional experiences.

**Fig. 1:**
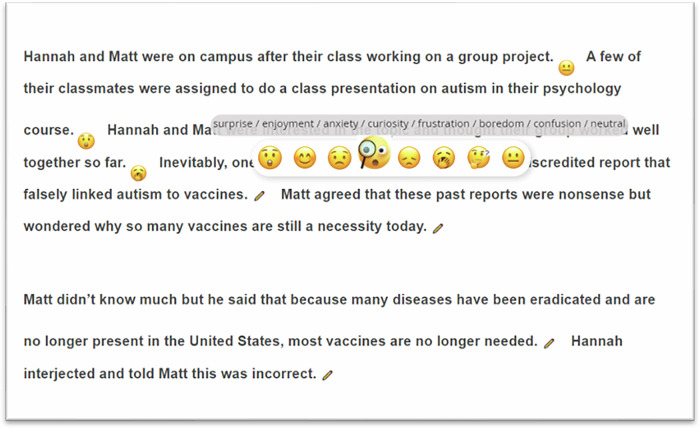
The *DynamicEmo* measure. This instrument enables participants to report emotions experienced while reading each sentence, thus allowing for granular data analysis. Also, multiple emotions and their dynamics can be captured within a single text.

The goal of this study is to investigate moment-to-moment epistemic emotions during reading of refutation texts and examine the influence of in-the-moment epistemic emotions on subsequent knowledge revision. This study builds on initial research conducted by Trevors and Kendeou^17^, who examined the effects of embedding different emotional content in refutation texts on knowledge revision, relying on the Knowledge Revision Components (KReC)^9^ framework. In the study, knowledge posttests and reading times were used to measure how undergraduate students processed refutation texts. The results showed that all refutation texts, regardless of their emotional content, facilitated learning as assessed by knowledge posttests. The reading time for correct-outcome sentences in negative refutation texts was shorter compared to non-refutation and positive refutation texts, implying that negative emotions are associated with increased salience and focused attention, thereby strengthening online revision. However, this study did not capture readers’ in-the-moment epistemic emotions. Therefore, we aim to both replicate and expand Trevors and Kendeou’s^17^ study in several ways: (a) We display the entire text on the screen, instead of sentence-by-sentence; (b) We employ a computer-based instrument (*DynamicEmo*) (see Fig. 1) to gauge participants’ emotional responses to each sentence as they read, allowing us to gain insight into the emotional processes involved in refutation texts; (c) We expand the population to all adults not only undergraduate university students. This enables us to assess the effectiveness of refutation texts across a more diverse population (e.g., ages, educational levels); and (d) We directly test the impact of experienced epistemic emotions during reading of critical correct-outcome sentences in different refutation texts on subsequent knowledge revision.

In the following sections, we first situate the current work within the KReC framework. Subsequently, we discuss the role of emotions in the processing of refutation information.

The Knowledge Revision Components (KReC) framework proposed by Kendeou and O’Brien^9^ elucidates the cognitive mechanisms of revision during reading of refutation texts. In the context of KReC, knowledge revision is seen as the process of minimizing the reactivation of previously encoded misconceptions. To facilitate this, refutational texts are deliberately designed to present incorrect statements (e.g., “developing immunity naturally from diseases is better than vaccine-acquired immunity”) that stimulate cognitive conflict. By placing accurate information (e.g., “natural infections may cause lifelong disability or death”) alongside the incorrect statements, the texts encourage competition between conflicting ideas. This approach is based on the view that misconceived knowledge is continuously revised rather than completely erased^18^. The knowledge revision process can be elucidated through three core principles— *coactivation*, *integration*, and *competing activation—* which are integral to effective revision^9^. Specifically. The *coactivation* principle posits that correct information needs to be simultaneously presented with the misconception^9^. In this case, simultaneous activation allows the *integration* principle to take place, in which the newly encoded information is integrated with the previously encoded false knowledge within the same mental model. This opens up the possibility of updating long-term memory representations to take into account new information^9^. Lastly, the *competing activation* principle illuminates that as the amount of newly encoded correct information escalates, it gradually dominates the integrated network of the information, consequently reducing the influence of previously acquired incorrect information^9^. Kendeou et al.^18^ used think-aloud and reading time measures to examine the process theorized within the KReC framework. They found more cognitive conflict and comprehension monitoring were verbally reported by readers after reading the refutation and explanation sections in refutation texts, providing evidence for the *coactivation* and *integration* principles. Furthermore, reduced cognitive conflict and increased inference making were reported after reading the correct outcome sentences, suggesting that the highly interconnected explanation in the refutation texts dominates the representation, thus reducing interference from prior misconceptions. This finding provided evidence for the *competing activation* principle. Also, faster reading times observed for the correct outcome sentence in refutation texts further support this^18^. The refutation approach has been implemented not only in textual formats but also as multimodal interventions (e.g., video-based messages)^19^. Moreover, its effectiveness has been demonstrated in promoting both short-term and long-term knowledge revision^20^. While the cognitive aspect of how people process refutation texts is widely understood, there has been less focus on scrutinizing how emotions are experienced during the reading of different sections of those texts and how these emotions influence knowledge revision.

Emotions associated with epistemic or knowledge-generating aspects of cognitive activities are called “epistemic emotions”. For instance, encountering cognitive incongruity may trigger emotions such as surprise and curiosity^21^. The epistemically-related emotion scale was developed to measure emotions that arise during epistemic cognitive activities, including surprise, curiosity, enjoyment, confusion, anxiety, frustration, and boredom^22^. From the perspective of the dimensional structure of emotion, these emotions can be categorized based on their valence and level of activation, arranging them within a two-dimensional circular space of pleasantness and activation (cf. circumplex models of affect)^23,24^. Curiosity and enjoyment are regarded as positive activating emotions, while confusion, frustration, and anxiety are seen as negative activating emotions. Boredom, on the other hand, is considered a negative deactivating emotion. Surprise is considered as an activating emotion, but its valence remains uncertain^22,25^. Regarding the link between emotions and knowledge revision, the Cognitive Reconstruction of Knowledge Model (CRKM)^26^ integrated the “hot” constructs (e.g., motivational factors) into conceptual change. Specifically, the interaction between the learner’s characteristics and the message characteristics affects the depth of cognitive engagement, ultimately influencing the likelihood of conceptual change. One effective approach involves using refutation texts to enhance situational interest, where learners often experience positive emotions (e.g., enjoyment), which boost cognitive engagement and thus facilitate knowledge revision^27^. On the other hand, the emotions experienced by readers could also reflect their attitudes or thoughts towards the content^28^, thereby providing a lens through which to understand the outcome of the knowledge revision process. For example, negative emotions and perceived threat may arise when individuals encounter refutation information that contradicts their own identity, resulting in the failure of refutation texts to revise misconceptions^14^. Relatedly, it is theorized that negative epistemic emotions may hinder the recall of essential refutation information and inhibit the processing of its content or the change of pre-existing attitudes^16^.

**Fig. 2:**
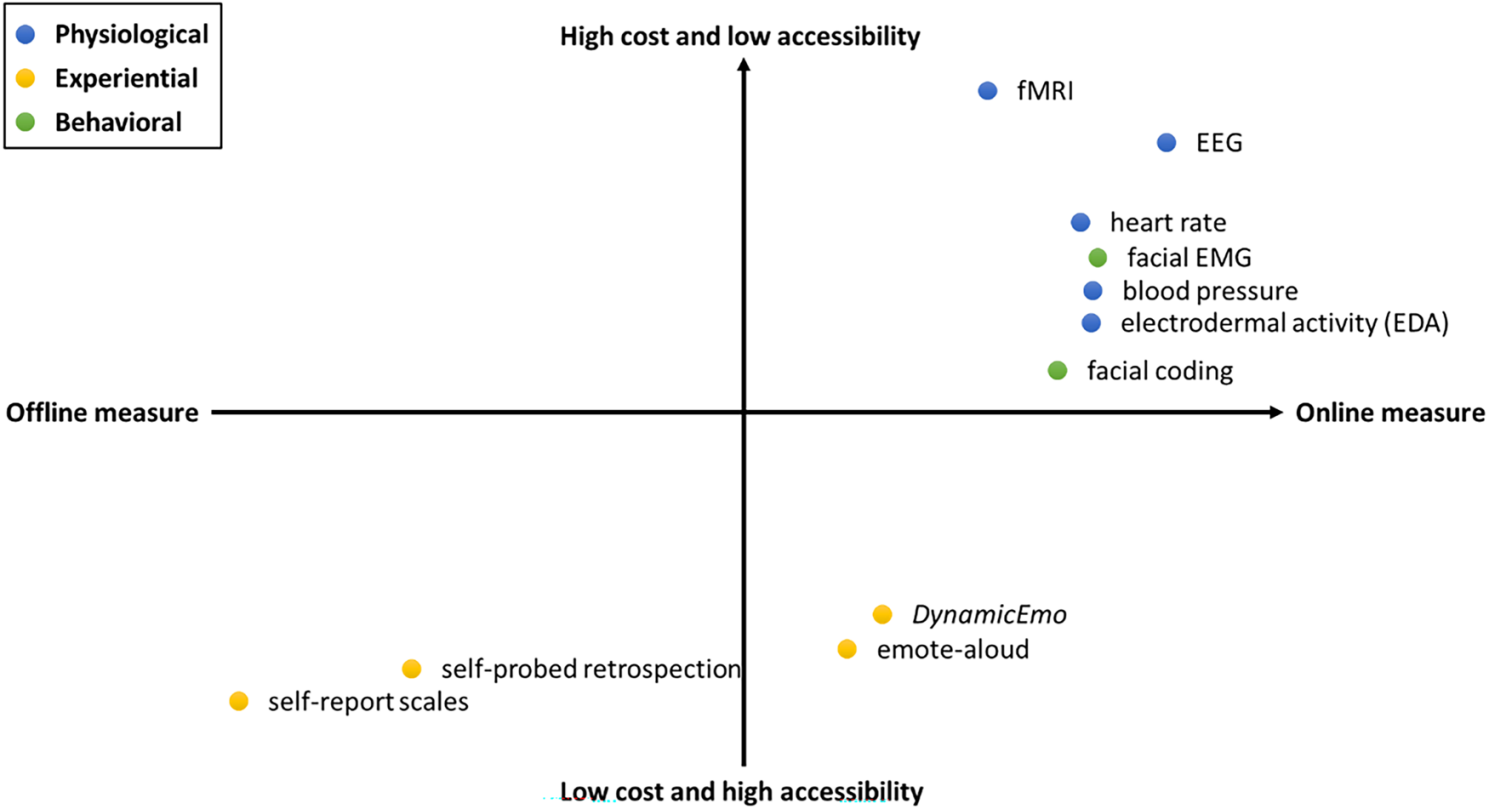
Measures of emotion in reading. These measures are assessed based on types (experiential, physiological, and behavioral), dynamics (online and offline methods), and considerations of cost and accessibility.

To assess emotions experienced by readers during interaction with texts, various methods can be employed, including *physiological*, *experiential*, and *behavioral* measures^29^. These measures can also be classified as either online (i.e., data collection or measurement that is conducted simultaneously with the phenomenon being studied, such as during reading) or offline (i.e., data collected after the phenomenon has already occurred, such as after reading) measures. Considering their types (experiential, physiological, and behavioral), dynamics (online and offline methods), and costs/accessibility, we provide an overview of measures of emotion in reading (Fig. 2). *Physiological* measures include data from neuroimaging techniques like functional magnetic resonance imaging (fMRI) and electroencephalography (EEG) for the central nervous system (CNS), and peripheral physiological measures include data from heart rate and skin conductance for autonomic nervous system activity (ANS). For instance, Jheng et al.^30^ used an electrodermal activity (EDA) measure to investigate emotional processes when reading engaging stories and dry expository texts. Although these measures can capture real-time processes in response to affective stimuli, the accessibility and costs might limit their application. *Experiential* measures include self-report scales like the Positive and Negative Affect Schedule (PANAS)^31^ and the Self-Assessment Mannikin (SAM)^32^, and methods like self-probed retrospection (i.e., underline texts that evoke emotion during reading, and then describe associated emotions participants think they had after reading)^33,34^ or emote-aloud (i.e., say aloud what emotions participants experienced while reading)^35,36^. Though self-probed retrospection and emote-aloud approach offer more detailed emotional insights than self-report scales, they might be influenced by memory biases (in retrospection) or interrupt the fluent understanding of texts (in emote-aloud). Furthermore, *behavioral* responses (e.g., facial behavior), to textual stimuli eliciting emotional appraisal can be measured through facial electromyography (EMG) responses and the facial coding method. For example, ’t Hart et al.^37^ found increased corrugator supercilii activity when reading narratives with immoral acts (inducing negative affect) and reduced muscle activity with moral acts (inducing positive affect). Moreover, the *DynamicEmo*, developed by the authors allows participants to select an emoji for each sentence while reading (an online measure), providing a more cost-effective and accessible alternative to traditional physiological measures.

The current study examines the knowledge revision effects of (emotional) refutation texts on a broader demographic of adults. Importantly, we seek to offer a more dynamic and nuanced analysis of the epistemic emotions experienced during reading and identify how in-the-moment emotions impact knowledge revision. Specifically, this study is guided by the following research questions: (i) Do knowledge revision outcomes differ among standard, negative, and positive refutation and non-refutation texts? (ii) Do epistemic emotions experienced by participants differ when reading refutation texts containing different emotional content? (iii) How do in-the-moment emotions during reading correct-outcome sentences affect knowledge revision? Drawing on the Knowledge Revision Components (KReC) framework^9^, we hypothesized that (emotional) refutation texts would outperform non-refutation texts in promoting knowledge revision. Furthermore, we proposed that epistemic emotions experienced during the processing of different refutation texts can be gauged using the *DynamicEmo* measure. Additionally, we expected to observe significant differences in epistemic emotions between paragraphs with consistent and inconsistent information. Third, we examined the emotions experienced towards correct-outcome sentences, strategically placed following the incorrect statement and refutation explanation, right before the closing paragraph. These sentences function as the final and crucial corrective statements^36^. We speculated that negative epistemic emotions experienced in response to correct-outcome sentences are associated with lower levels of knowledge revision.

## Results

### Examining knowledge revision across refutation texts

**Fig. 3:**
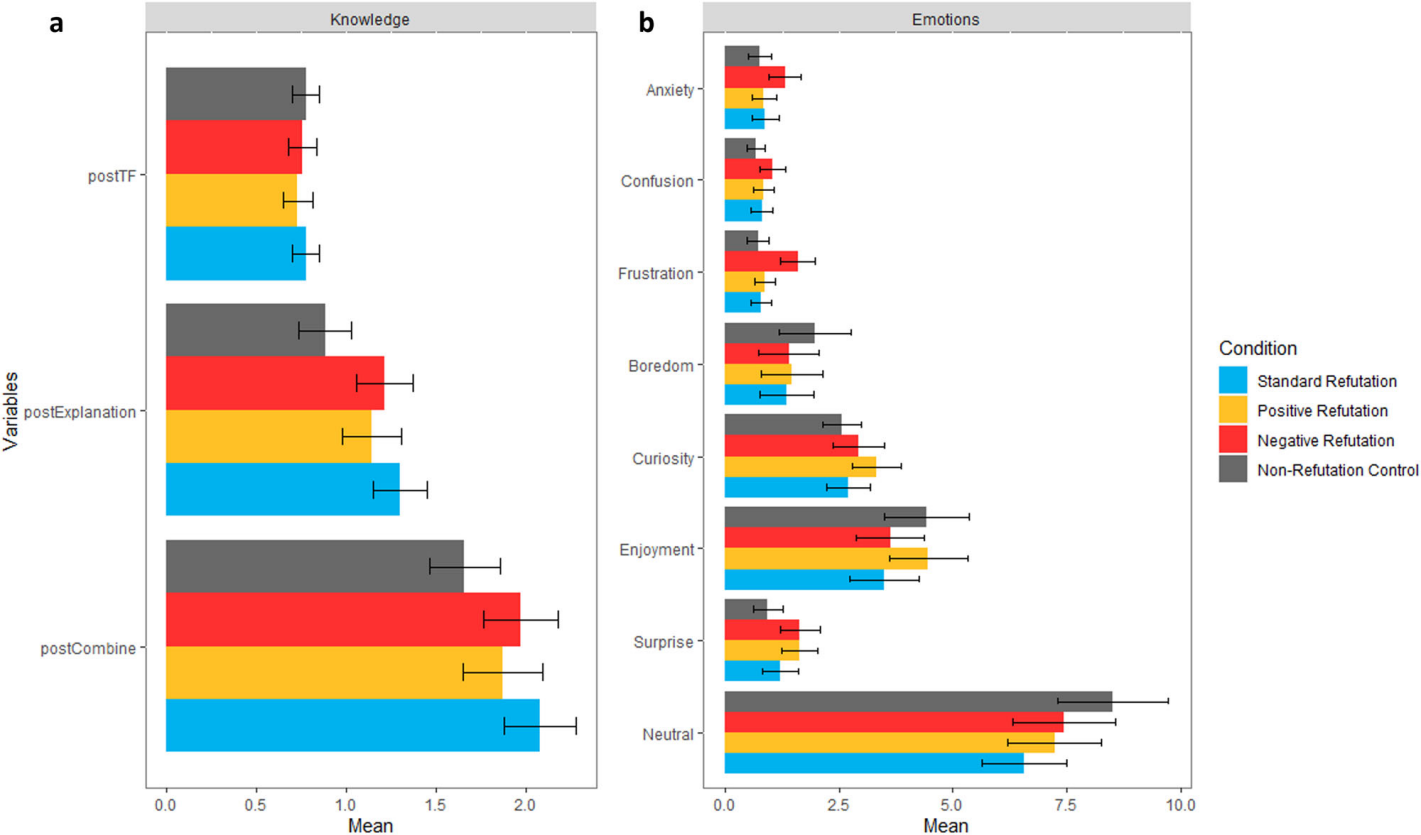
Descriptive analyses of knowledge posttest results and epistemic emotions. Bar graph showing means and 95% confidence intervals for **a** knowledge post-test scores and **b** counts of epistemic emotions measured through *DynamicEmo* across different text conditions.

We analyzed the effect of refutation texts on knowledge revision, measured by True/False questions (*postTF*), open-ended explanations (*postExplanation*), and combined question scores (*postCombine*). Mixed-effects models were used to assess differences in knowledge posttest across text conditions. Descriptive analyses for all variables are in Fig. 3a, b, while descriptive statistics and mixed-effects models for the knowledge posttest are in Supplementary Table 1 and Supplementary Table 2. Post-hoc comparisons for mixed models (Table 1) showed no significant differences between text conditions for the *postTF*. However, results of the *postExplanation* indicated participants gained significantly higher scores in open-ended explanation questions when exposed to standard refutation (*b* = 1.40, *p* < 0.001), positive refutation (*b* = 1.00, *p* = 0.005), and negative refutation texts (*b* = 1.05, *p* = 0.002) compared to non-refutation texts. In terms of the *postCombine*, participants who read standard refutation texts (*b* = 1.15, *p* < 0.001), positive refutation (*b* = 0.82, *p* = 0.02), and negative refutation texts (*b* = 0.81, *p* = 0.02) attained significantly higher overall scores compared to those who read non-refutation texts. Moreover, an effect of age on knowledge revision was observed. As age increased, participants’ knowledge posttest scores decreased, with each additional year of age associated with a 0.04-unit reduction in both *postExplanation* (*b* = −0.04, *p* = 0.005) and *postCombine* (*b* = −0.04, *p* = 0.007) (Supplementary Table 2).

**Table 1:** Parameter estimates of the multiple comparisons of means for text conditions and educational level

	*postTF*				*postExplanation*				*postCombine*			
	*B*	*SE*	*Z*	*pr(>|z* | *)*	*B*	*SE*	*z*	*pr(>|z* | *)*	*B*	*SE*	*z*	*pr(>|z* | *)*
Standard vs. Positive	0.31	0.42	0.75	1.00	0.39	0.30	1.32	1.00	0.33	0.28	1.16	1.00
Standard vs. Negative	0.38	0.43	0.88	1.00	0.35	0.30	1.19	1.00	0.34	0.28	1.22	1.00
Standard vs. Non-Refutation	0.28	0.44	0.64	1.00	1.40	0.30	4.67	<0.001	1.15	0.28	4.10	<0.001
Positive vs. Negative	0.07	0.42	0.16	1.00	−0.04	0.30	−0.15	1.00	0.01	0.28	0.05	1.00
Positive vs. Non-Refutation	−0.03	0.43	−0.07	1.00	1.00	0.30	3.36	0.005	0.82	0.28	2.94	0.02
Negative vs. Non-Refutation	−0.10	0.44	−0.22	1.00	1.05	0.29	3.57	0.002	0.81	0.27	2.94	0.02
High school- Technical/community college	0.63	0.63	1.00	1.00	−0.03	0.50	−0.07	1.00	0.23	0.49	0.47	1.00
High school- Undergraduate	−0.32	0.69	−0.46	1.00	−0.69	0.53	−1.29	1.00	−0.52	0.52	−1.00	1.00
High school- Graduate	−0.03	1.08	−0.02	1.00	−0.41	0.78	−0.53	1.00	−0.25	0.77	−0.33	1.00
High school- Doctorate	0.50	1.78	0.28	1.00	0.52	1.58	0.33	1.00	0.47	1.48	0.32	1.00
Technical/community college- Undergraduate	−0.95	0.69	−1.38	1.00	−0.65	0.53	−1.24	1.00	−0.75	0.52	−1.44	1.00
Technical/community college- Graduate	−0.66	1.10	−0.60	1.00	−0.38	0.80	−0.48	1.00	−0.48	0.79	−0.61	1.00
Technical/community college- Doctorate	−0.14	1.76	−0.08	1.00	0.55	1.56	0.35	1.00	0.24	1.46	0.16	1.00
Undergraduate- Graduate	0.29	1.14	0.26	1.00	0.27	0.83	0.33	1.00	0.27	0.81	0.34	1.00
Undergraduate- Doctorate	0.81	1.75	0.47	1.00	1.20	1.54	0.78	1.00	0.99	1.44	0.69	1.00
Graduate - Doctorate	0.52	2.02	0.26	1.00	0.93	1.71	0.54	1.00	0.72	1.62	0.44	1.00

### Emotions experienced in reading refutation texts

To investigate epistemic emotions across different text conditions, mixed-effects models were used to analyze emotion data at the *text* level. Descriptive statistics and mixed-effects models for epistemic emotions are detailed in Supplementary Table 3 and Supplementary Table 4. Post-hoc comparisons between text conditions are shown in Table 2. The *anxiety* levels of participants were significantly higher when reading negative refutation texts compared to positive refutation (*b* = −0.47, *p* = 0.01), standard refutation (*b* = −0.54, *p* = 0.001) and non-refutation texts (*b* = 0.65, *p* < 0.001). Likewise, participants indicated significantly heightened levels of *frustration* when exposed to negative texts compared to positive refutation (*b* = −0.60, *p* < 0.001), standard refutation (*b* = −0.66, *p* < 0.001) and non-refutation texts (*b* = 0.84, *p* < 0.001). Additionally, participants reported significantly greater *confusion* when exposed to the negative refutation than the non-refutation condition (*b* = 0.46, *p* = 0.02). Furthermore, the *boredom* levels of participants reading non-refutation texts were significantly higher than those of participants reading standard refutation (*b* = −0.40, *p* = −0.004), positive refutation (*b* = −0.31, *p* = 0.04) and negative refutation texts (*b* = −0.41, *p* = 0.003). Similarly, participants in the non-refutation condition rated significantly more *neutral* emotions compared to those in the standard refutation (*b* = −0.26, *p* < 0.001), positive refutation (*b* = -.16, *p* = .01) and negative refutation conditions (*b* = −0.16, *p* = 0.02). Moreover, the *curiosity* levels of participants exposed to positive refutation texts were significantly higher than those of participants reading standard refutation (*b* = −0.23, *p* = 0.03) and non-refutation texts (*b* = 0.28, *p* = 0.006). Participants rated significantly higher levels of *enjoyment* when reading positive refutation (*b* = −0.25, *p* = 0.005) and non-refutation texts (*b* = −0.20, *p* = 0.04) compared to standard refutation texts. In addition, participants reported significantly higher levels of *surprise* when exposed to positive refutation texts than when exposed to standard refutation texts (*b* = −0.37, *p* = 0.02). Also, participants experienced significantly more surprise when reading both positive refutation (*b* = 0.63, *p* < 0.001) and negative refutation texts (*b* = 0.52, *p* < 0.001) compared to non-refutation texts.

**Table 2:** Parameter estimates of the multiple comparisons of means for text conditions

	Anxiety				Boredom			
	*B*	*SE*	*Z*	*pr(>|z* | *)*	*B*	*SE*	*z*	*pr(>|z* | *)*
Standard vs. Positive	−0.07	0.16	−0.44	1.00	−0.09	0.13	−0.74	1.00
Standard vs. Negative	−0.54	0.15	−3.69	0.001	0.01	0.13	0.04	1.00
Standard vs. Non-Refutation	0.11	0.16	0.69	1.00	−0.40	0.12	−3.40	0.004
Positive vs. Negative	−0.47	0.14	−3.30	0.01	0.10	0.13	0.78	1.00
Positive vs. Non-Refutation	0.18	0.16	1.11	1.00	−0.31	0.11	−2.75	0.04
Negative vs. Non-Refutation	0.65	0.15	4.39	<0.001	−0.41	0.12	−3.48	.003
	**Confusion**				**Curiosity**			
	*B*	*SE*	*Z*	*pr(>|z* | *)*	*B*	*SE*	*z*	*pr(>|z* | *)*
Standard vs. Positive	0.01	0.16	0.08	1.00	−0.23	0.08	−2.77	0.03
Standard vs. Negative	−0.23	0.15	−1.53	0.76	−0.07	0.09	−0.84	1.00
Standard vs. Non-Refutation	0.23	0.17	1.39	0.99	0.05	0.09	0.50	1.00
Positive vs. Negative	−0.24	0.15	−1.63	0.62	0.16	0.08	1.97	.29
Positive vs. Non-Refutation	0.22	0.17	1.33	1.00	0.28	0.09	3.29	.006
Negative vs. Non-Refutation	0.46	0.16	2.93	0.02	0.12	0.09	1.35	1.00
	**Enjoyment**				**Frustration**			
	*B*	*SE*	*Z*	*pr(>|z* | *)*	*B*	*SE*	*z*	*pr(>|z* | *)*
Standard vs. Positive	−0.25	0.07	−3.36	0.005	−0.06	0.16	−0.36	1.00
Standard vs. Negative	−0.08	0.08	−1.07	1.00	−0.66	0.14	−4.68	<0.001
Standard vs. Non-Refutation	−0.20	0.07	−2.72	0.04	0.18	0.17	1.09	1.00
Positive vs. Negative	0.17	0.07	2.28	0.14	−0.60	0.14	−4.45	<0.001
Positive vs. Non-Refutation	0.05	0.07	0.68	1.00	0.24	0.16	1.47	0.85
Negative vs. Non-Refutation	−0.12	0.07	−1.63	0.61	0.84	0.14	5.81	<0.001
	**Neutral**				**Surprise**			
	*B*	*SE*	*Z*	*pr(>|z* | *)*	*B*	*SE*	*z*	*pr(>|z* | *)*
Standard vs. Positive	−0.09	0.06	−1.68	0.56	−0.37	0.13	−2.94	0.02
Standard vs. Negative	−0.10	0.06	−1.85	0.39	−0.26	0.12	−2.13	0.20
Standard vs. Non-Refutation	−0.26	0.05	−4.77	<0.001	0.26	0.14	1.85	0.39
Positive vs. Negative	−0.01	0.05	−0.16	1.00	0.11	0.12	0.94	1.00
Positive vs. Non-Refutation	−0.16	0.05	−3.12	0.01	0.63	0.13	4.77	<0.001
Negative vs. Non-Refutation	−0.16	0.05	−2.98	0.02	0.52	0.13	3.95	<0.001

Regarding analyzing emotions at the *paragraph* level, descriptive statistics (counts and percentage) for *activating* and *deactivating* emotions, are shown in Supplementary Table 5. Supplementary Fig. 1 shows the percentage of activating (or deactivating) emotions reported in a specific paragraph within a given text, and it demonstrates how these percentage values differ across text conditions. Intuitively, there was a distinct difference in reported activating or deactivating emotions between the refutation text and non-refutation text in paragraphs 2 and 3 compared to paragraph 1.

The results of mixed models regarding their post-hoc comparisons between paragraphs are shown in Table 3. Significant interactions were observed between text condition and paragraph. In paragraph 2 (misinformation description), differences in activating emotions were found between negative refutation and non-refutation (*b* = 1.58, *p* < 0.001), non-refutation and positive refutation (*b* = 0.62, *p* < 0.001), positive and standard refutation text (*b* = 1.31, *p* = 0.03) and negative and standard refutation text (*b* = 1.28, *p* < 0.05). In paragraph 3 (refutation section), significant differences in deactivating emotions were found between negative refutation and non-refutation (*b* = 0.29, *p* < 0.001), non-refutation and positive refutation (*b* = 4.23, *p* < 0.001), and non-refutation and standard refutation text (*b* = 3.87, *p* < 0.001). Also, in paragraph 4 (continuation), activating emotions varied significantly between negative refutation and non-refutation (*b* = 1.63, *p* < 0.001), non-refutation and positive refutation (*b* = 0.69, *p* < 0.001), negative and standard refutation (*b* = 1.86, *p* < 0.001) and positive and standard refutation text (*b* = 1.64, *p* < 0.001). Deactivating emotions also showed significant differences between negative refutation and non-refutation (*b* = 1.37, *p* = 0.005), non-refutation and positive refutation (*b* = .66, *p* < 0.001), and positive and standard refutation text (*b* = 1.38, *p* = 0.002). As expected, no significant differences were observed in paragraphs 1 (introduction) and 5 (closing), which were identical across conditions.

**Table 3:** Parameter estimates of the multiple comparisons of means for paragraphs

	Activating emotions				Deactivating emotions			
Paragraph 1	*B*	*SE*	*Z*	*pr(>|z* | *)*	*B*	*SE*	*z*	*pr(>|z* | *)*
Negative vs. Non-Refutation	1.02	0.09	0.24	0.99	0.97	0.09	−0.39	0.98
Negative vs. Positive	0.99	0.09	−0.12	1.00	1.02	0.09	0.24	1.00
Negative vs. Standard	1.05	0.09	0.52	0.95	0.94	0.08	−0.74	0.88
Non-Refutation vs. Positive	0.97	0.08	−0.37	0.98	1.06	0.10	0.63	0.92
Non-Refutation vs. Standard	1.03	0.09	0.28	0.99	0.97	0.09	−0.34	0.99
Positive vs. Standard	1.06	0.09	0.64	0.92	0.92	0.08	−0.97	0.77
**Paragraph 2**	*B*	*SE*	*Z*	*pr(>|z* | *)*	*B*	*SE*	*z*	*pr(>|z* | *)*
Negative vs. Non-Refutation	1.58	0.16	4.46	<0.001	0.83	0.11	−1.36	0.53
Negative vs. Positive	0.98	0.09	−0.26	0.99	1.03	0.15	0.21	1.00
Negative vs. Standard	1.28	0.12	2.57	<0.05	1.78	0.30	3.41	0.004
Non-Refutation vs. Positive	0.62	0.06	−4.70	<0.001	1.24	0.17	1.56	0.40
Non-Refutation vs. Standard	0.81	0.09	−1.91	0.22	2.15	0.36	4.62	<0.001
Positive vs. Standard	1.31	0.13	2.82	0.03	1.73	0.30	3.19	<0.01
**Paragraph 3**	*B*	*SE*	*Z*	*pr(>|z* | *)*	*B*	*SE*	*z*	*pr(>|z* | *)*
Negative vs. Non-Refutation	1.03	0.09	0.29	0.99	0.29	0.03	−10.68	<0.001
Negative vs. Positive	0.96	0.08	−0.47	0.97	1.22	0.19	1.32	0.55
Negative vs. Standard	0.99	0.09	−0.15	1.00	1.12	0.17	0.75	0.88
Non-Refutation vs. Positive	0.94	0.08	−0.76	0.87	4.23	0.54	11.34	<0.001
Non-Refutation vs. Standard	0.96	0.08	−0.44	0.97	3.87	0.48	10.95	<0.001
Positive vs. Standard	1.03	0.09	0.32	0.99	0.91	0.15	−0.57	0.94
**Paragraph 4**	*B*	*SE*	*Z*	*pr(>|z* | *)*	*B*	*SE*	*z*	*pr(>|z* | *)*
Negative vs. Non-Refutation	1.63	0.15	5.39	<0.001	1.37	0.13	3.33	0.005
Negative vs. Positive	1.13	0.09	1.52	0.42	0.90	0.08	−1.26	0.59
Negative vs. Standard	1.86	0.18	6.48	<0.001	1.24	0.11	2.34	0.09
Non-Refutation vs. Positive	0.69	0.06	−3.90	<0.001	0.66	0.06	−4.53	<0.001
Non-Refutation vs. Standard	1.14	0.12	1.23	0.60	0.91	0.09	−0.99	0.76
Positive vs. Standard	1.64	0.16	5.04	<0.001	1.38	0.13	3.55	0.002
**Paragraph 5**	*B*	*SE*	*Z*	*pr(>|z* | *)*	*B*	*SE*	*z*	*pr(>|z* | *)*
Negative vs. Non-Refutation	0.86	0.14	−0.96	0.77	1.06	0.14	0.43	0.97
Negative vs. Positive	0.96	0.16	−0.23	1.00	0.99	0.13	−0.06	1.00
Negative vs. Standard	1.20	0.21	1.05	0.72	0.85	0.10	−1.29	0.57
Non-Refutation vs. Positive	1.12	0.18	0.73	0.89	0.94	0.12	−0.49	0.96
Non-Refutation vs. Standard	1.40	0.24	1.99	0.19	0.81	0.10	−1.70	0.32
Positive vs. Standard	1.25	0.22	1.27	0.58	0.86	0.11	−1.22	0.62

### Effect of in-the-moment reported emotions towards the correct-outcome sentence on knowledge revision

**Fig. 4:**
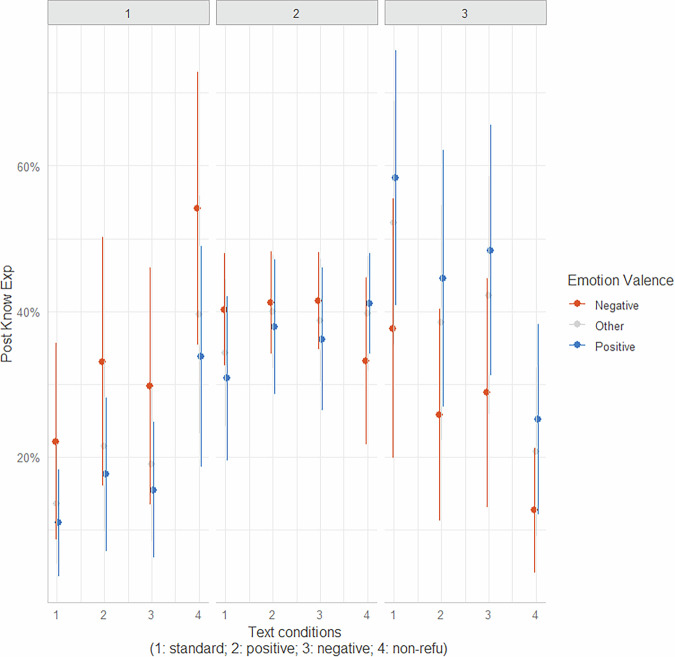
Predicted probability of *postExplanation* by *text conditions* and *emotions.* The plot illustrates the estimated probability of knowledge posttest scores as a function of experienced emotions toward the correct-outcome sentence under different text conditions.

The mean knowledge revision (*postExplanation*) scores for different text conditions and experienced emotions towards the correct-outcome sentences are presented in Supplementary Table 6. The analyses revealed main effects of both text conditions and participants’ experienced emotions toward the correct-outcome sentences on their scores of the knowledge posttest (*postExplanation*). Specifically, *postExplanation* scores were significantly lower when participants experienced negative emotions toward the correct-outcome sentences compared to positive emotions (*b* = −0.84, *p* < 0.05). Additionally, there were consistently lower scores of knowledge posttest observed during the reading of non-refutation texts compared to standard refutation (*b* = 1.43, *p* < 0.001), positive refutation (*b* = 0.87, *p* = 0.03), and negative refutation texts (*b* = 1.03, *p* = 0.004) (Supplementary Table 7 and Supplementary Table 8, and Fig. 4).

## Discussion

This study replicated the effect of refutation texts on knowledge revision, measured by *open-ended explanation* and *combined True/False and explanation question* scores, revealing significant differences between text conditions. However, no significant differences were found for *True/False question* scores, indicating that while refutation text type did not notably affect participants’ performance on these binary questions, it did impact their performance on explanation quality and overall understanding. The lack of differentiation in *True/False* scores may be due to their limited sensitivity to nuanced effects, potential guessing, and a ceiling effect from widespread vaccination-related information during the COVID-19 pandemic. Indeed, our data reflected this trend, with 61% correct responses on pretest True/False questions compared to a lower average prior knowledge score of 48% in the original study. The open-ended explanation questions are likely to be more sensitive to the depth of knowledge and understanding compared to the binary True/False questions in the current context.

In this study, we found that when participants were exposed to (standard, positive, negative) refutation texts, they gained significantly higher scores in knowledge posttests (both scores from explanation questions and scores combining explanation and True/False questions) after controlling prior knowledge compared to when exposed to non-refutation texts. Our study, extending to a more diverse participant pool, effectively reproduced the findings of the original research. It is worth noting that a significant age effect was also observed on knowledge revision. In particular, older participants demonstrated lower knowledge posttest scores, suggesting that age may have an impact on the ability to integrate information and revise misconceptions. This finding is particularly noteworthy, especially in light of a review paper that reported approximately 70% of the refutation studies using college undergraduates as their sample population^7^. The current study indicated that refutation prompts immediate knowledge revision across a broader demographic spectrum. This may align with findings from research on older adults, where exposure to corrective materials led to an immediate decline in misinformation adherence^38^. In addition, refutation texts, irrespective of emotional content, are more effective in enhancing knowledge revision compared to non-refutation texts. Prior research about the debunking of misconceptions revealed that the format or tone of correction holds less sway, while the provision of corrective information is crucial in debunking misinformation and encouraging individuals to engage with or seek further corrective information^39,40^. In line with this, the current study reaffirmed the importance of presenting refutational explanation information to effectively counter misconceptions.

In the analysis of the experienced emotions at the *text* level, our findings supported the hypothesis on the pleasantness/valence dimension. Consistent with expectations, our results showed that negative refutation texts induced significantly higher levels of negative activating emotions, such as *anxiety and frustration*, compared to positive refutation, standard refutation, and non-refutation texts. Moreover, negative refutation texts were associated with greater *confusion* compared to non-refutation texts. On the other hand, positive refutation texts elicited notably higher levels of *curiosity* than both standard refutation and non-refutation texts, as well as greater *enjoyment* than standard refutation. Results showed that participants exposed to non-refutation texts reported heightened levels of *boredom* and *neutral* emotions compared to those exposed to standard refutation, positive refutation, and negative refutation texts. Furthermore, participants reported significantly lower levels of activating emotion (*surprise*) when exposed to non-refutation texts compared to positive refutation and negative refutation texts.

In the analysis of the experienced emotions at the *paragraph* level, we observed differences in less activating and more deactivating emotions generally when reading non-refutation compared to refutation texts, particularly evident in paragraph 2 (misinformation description), paragraph 3 (refutation section), and paragraph 4 (continuation). Conversely, there were no significant differences in the numbers of activating emotions or deactivating emotions reported between text conditions in paragraph 1 (introduction) and paragraph 5 (closing). This suggests that the presentation of inconsistent information (e.g., presenting misinformation followed by directly refute it) in emotional refutation texts did elicit activating epistemic emotions (such as curiosity, confusion, frustration, enjoyment, anxiety, and surprise). Also, refutation explanations did repress deactivating epistemic emotions (such as boredom and neutral).

The correct-outcome sentences played a crucial role as the final affirmation of corrective information within the refutation texts. In our study, we directly investigated the impact of emotions reported towards these correct-outcome sentences on knowledge revision. The results revealed that scores of knowledge explanations were significantly lower when participants reported negative emotions towards the correct-outcome sentences, compared to those reported positive emotions. This finding may relate to previous research indicating that post-knowledge among readers engaged in the knowledge revision process was positively predicted by surprise and negatively predicted by hopelessness^41^. One possible explanation is that negative emotions may occur when the newly encoded correct information has still not yet gained enough strength to outweigh the outdated misconceptions, leading to resistance. In this case, negative emotions may reflect cognitive resistance to the new information, as the readers struggle to reconcile it with existing knowledge. These results highlight the significant role of emotions in the revision process, suggesting that epistemic emotions—particularly those linked to the final, correct-outcome information—may be a signal of whether individuals will eventually revise their knowledge.

Our study involved a more diverse participant sample and successfully replicated the results, indicating that refutation is an effective approach to promote knowledge revision, yet it is important to note that an immediate knowledge posttest was used. Previous research showed that after a week’s delay, participants’ endorsement of misinformation reverted to levels observed prior to exposure to refutations^38^. Similarly, participants’ belief in incorrect information increased two days after a correction^42^. Future studies can look into delayed post-tests to investigate how refutation texts containing emotional language might enhance the memorability of certain information, thereby improving memory retention for specific details, even over time.

This study primarily examined the impact of emotional content within refutation texts on knowledge revision. However, it did not thoroughly investigate other psychological factors related to the understanding of vaccination topics, such as personal beliefs, trust in the health care system, and belief in vaccination conspiracy theories, which could also influence the results^43,44^. Furthermore, although our original intention was to have a vaccine hesitancy scale into the study, it was administered in the post-survey to avoid priming participants and influencing their emotional experience while reading the texts. However, responses to scale items may be influenced by the knowledge participants acquired while reading. As a result, we chose to exclude this aspect from our analysis. We may seek to explore self-concepts and personal beliefs in a separate study. Future research could explore how these psychological factors interact with emotional responses to different emotional refutation texts and subsequently influence knowledge revision. For example, individuals who distrust the healthcare system may be more sensitive to refutation texts containing emotional language and may show selective processing of vaccination refutation information to align with their pre-existing attitudes, which in turn impact knowledge revision.

Furthermore, it’s important to recognize that different measures may capture different facets of the phenomenon under investigation. In the original study, reading time and knowledge tests were used. Online reading time may reflect the ease of integrating new information into memory and the offline measure of declarative knowledge may offer insight into retrieval processes^17^. In the current study, we shifted our focus towards experienced emotion as an online measure using *DynamicEmo*. By examining participants’ real-time emotional experiences of the critical sentence, we attempted to deepen our understanding of how these emotions might be linked to declarative knowledge. Future studies could consider integrating additional online measures, such as eye tracking, to further tap into cognitive processes underlying refutation texts, and how emotional experiences and cognitive processes interact in knowledge revision.

Moreover, the *DynamicEmo* measure is quick and intuitive, akin to actions commonly performed on social media, where individuals express reactions spontaneously. It may facilitate the identification of patterns in emotions during reading processes and their potential correlation with learning outcomes. Relatedly, given its ability to capture emotions in real-time, further studies could use this measure to monitor the emotions experienced by readers, thus providing scaffolding. For instance, in our study, we found that negative emotions reported by readers during the processing of critical sentences negatively predicted knowledge revision. Beyond theoretical implications, these results have practical relevance for educational settings. If the system detects such emotions while a participant reads a correct-outcome sentence in a refutation text, it could immediately offer additional supportive resources. For example, it might provide explanatory notes for those who prefer analytical thinking or display a pop-up discussion window where learners can express their doubts or opinions to educators, while automatically logging their thoughts for educators to build up further discussion and resolve.

In sum, building upon previous work by Trevors and Kendeou^17^, our study extended to a more diverse participant pool and successfully replicated the original findings. Our results confirmed that refutation texts, regardless of emotional content, are more effective in enhancing knowledge revision than non-refutation texts. Moreover, this study advanced the dynamic measurement of emotions while reading (refutation) texts by introducing a novel measure, called *DynamicEmo*. It captured experienced moment-to-moment emotions, enabling analysis at the text, paragraph, and sentence levels. At the text level, negative refutation texts generally induced more negative emotions, while positive refutation texts evoked more positive emotions. At the paragraph level, the central sections of refutation texts presenting inconsistent information (misinformation and refutation explanation) stimulated more activating emotions or less deactivating emotions compared to equivalent sections in non-refutation texts. Lastly, our findings revealed that negative emotions triggered by critical sentences (correct-outcome sentences) serve as negative predictors of knowledge explanations, emphasizing their importance in predicting knowledge revision. In conclusion, this study proves the robustness of the refutation effect across a broader age demographic. Additionally, we contributed a measure for epistemic emotions during the online reading process and underscored the significance of emotions towards correct-outcome sentences in refutation texts, as it predicts knowledge outcomes.

## Methods

### Participants

This study was preregistered on the AsPredicted (#140378). A statistical power analysis (GPower3.1.9.4) was performed based on data from Trevors and Kendeou^17^ experiment 3 (*N* = 45; η^2^_p_ = 0.15), comparing knowledge posttest scores in different text conditions. Results indicated 56 participants are needed to achieve a power of.80 at a significance level α = 0.05. In this study, participants were recruited via *Prolific*. Fifty-six participants (*M* = 40.34 years, *SD* = 14.61 years; female = 35) with valid knowledge posttest responses were included in this study.

### Texts

Text materials, sourced from Trevors and Kendeou^17^, included four refutation types: standard refutation, positive refutation, negative refutation, and non-refutation. Each text corrected one of the eight common misconceptions about vaccines and was structured into five paragraphs: introduction, misinformation description, refutation explanations, continuation (either continuation or continuation-plus-emotion-reinstatement) and closing.

There are differences between (standard, positive, negative) refutation and non-refutation texts in the misinformation, refutation, and continuation paragraphs. In the former, a character directly expressed misinformation (e.g., “Brandon hadn’t gotten vaccinated but told Nicole that he thought it didn’t matter…He said that it was a safe technique to rely on the vaccines of others”), which is immediately corrected by the other character (e.g., “Nicole said this wasn’t true”) and followed by explanations (e.g., “Nicole explained that people who take advantage of herd immunity – “hiding in the herd” – choose the riskier option”) in refutation and continuation paragraphs. In contrast, in the latter (non-refutation texts), the character neither explicitly stated any false information nor is corrected by the other character. Moreover, the distinction between standard (neutral) and emotional (positive, negative) refutation texts lay in their emotional content. Further details are available on the OSF.

### Measures

*DynamicEmo* measure. We developed an online measure called the *DynamicEmo*, which instructed participants to select an emoji to indicate their emotional experience for each sentence of the refutation text (Fig. 1). This measure allows to gauge the dynamics of emotions experienced throughout the course of reading, as well as assess multiple emotions within a single text. *DynamicEmo* included seven epistemic emotions from the Epistemically-Related Emotion Scales^22^: surprise, curiosity, enjoyment, confusion, anxiety, frustration, and boredom. Participants could select “neutral” if they did not experience a strong or specific feeling toward specific sentences. In this study, we use single-item indicators, such as an emoji icon representing anxiety, to assess participants’ experienced anxiety. A similar approach, using single-item measures in the form of checklists, has been employed to assess affective states during interactions with e-learning systems^45^. Furthermore, existing literature indicates single-item measures can serve as psychometrically sound alternatives to multi-item scales, particularly when time constraints limit survey length^46^. These findings support the reliability of the approach adopted in this study.

#### Knowledge tests

Knowledge tests included both pretest and posttest measures, obtained from the original study^17^. The pretest assessed participants’ prior knowledge about eight common vaccine misconceptions using True/False questions. Participants received one point for a correct answer and zero points for an incorrect answer (score range 0−1). The posttest also comprised eight True/False questions (e.g., Vaccines work by causing the full-blown symptoms of the specified disease. False or True), followed by an open-ended explanation question (e.g., Please explain your answer: _____) for each item. The posttest had three metrics: knowledge posttest True/False questions (*postTF*) (score range 0−1), open-ended explanation questions (*postExplanation*) (score range 0−2, with 2 points awarded for a completely correct explanation, 1 point for a partially correct explanation, and 0 points for a completely incorrect explanation), and a metric combining the scores of both True/False questions and explanation questions (*postCombine*) (score range 0−3). To establish the reliability of the scoring process, two coders were involved in coding the explanations. The first author coded all responses, while a co-author independently coded a randomly selected 10% of explanations. Both coders adhered to a predefined coding manual and assessment rubric, ensuring consistency in score assignment. The coding instructions can be accessed on OSF. An inter-rater reliability analysis was performed, resulting in a Cohen’s Kappa (κ) of 0.83, reflecting strong agreement^47^.

### Procedure

All participants were presented with informed consent, and they could only proceed with the online experiment by clicking on the “consent to participate in this experiment voluntarily” button on the webpage. Participants first completed the knowledge pretest. Subsequently, eight texts were randomly assigned to each participant, including two standard (neutral) refutation, two positive refutation, two negative refutation, and two non-refutation texts. Participants were asked to read texts while reporting their experienced emotions toward each sentence with *DynamicEmo*. Afterwards, they completed the knowledge posttest for all texts, which were presented in a random order. Finally, they completed a demographic questionnaire covering items such as gender, highest education level, and vaccination experiences, as detailed in the original paper. Each participant received £7.50 for their participation. The ethics approval was obtained from the Ethics Committee for the Social Sciences and Humanities (EA SHW) of the University of Antwerp (ID SHW_20_62).

### Data analysis

The data were analyzed with cumulative link (CLMMs) and generalized linear (GLMM) mixed-effects models in R software (version 4.1.2, R Core Team, 2023). The data sets and scripts can be found on the OSF (Open Science Framework): https://osf.io/rwta8/?view_only=a40eb0f6228f46ca977fa6778b74b046.

#### Knowledge posttest

To test whether knowledge revision differed among refutation texts, mixed-effects models were applied to analyze the knowledge posttest, with *postTF*, *postExplanation*, and *postCombine* serving as dependent variables. Concurrently, *knowledge pretest (preTF)*, *text condition, participant’s age, and educational level* were considered as fixed effects, while *participant* and *text* were considered as random effects in the analysis. Since *postTF* is a binary outcome variable, we fitted a binomial GLMM model using the *glmer()* function in the *lme4* package (version 1.1–28)^48^. Both *postExplanation* and *postCombine* are ordinal data. We thus analyzed them with cumulative link mixed model (CLMMs) using the *ordinal* package (version 2022.11–16)^49^. The multiple comparisons of means, adjusted for the Bonferroni correction, were performed using the *emmeans* package (version 1.7.4–1)^50^, ensuring appropriate control of family-wise error rate across all tests conducted.

#### Experienced emotions

To test the extent to which epistemic emotions experienced by participants vary with different conditions, mixed-effects models were used with each epistemic *emotion* as the dependent variable. The *text condition* was considered as the fixed effect, while both *participant* and *text* were considered as random effects. Count data of experienced emotions were fitted with Poisson GLMM model using the *glmer()* function in the *lme4* package (version 1.1–28)^48^. Additionally, to examine the differences in emotions across text conditions while participants read various *paragraphs*, we conducted two Poisson GLMM models with counts of *activating* and *deactivating* emotions as the dependent variables. The models included fixed effects for *text condition*, *paragraph*, and *their interaction*, as well as random effects for *participant* and *text*.

#### Emotions in predicting knowledge revision

To investigate whether the experienced emotions towards the correct-outcome sentences and the reading of the different texts could predict knowledge revision, mixed-effects models were used with *postExplanation* as the dependent variable. The *emotions towards the correct-outcome sentences* and *text condition* were entered as fixed effects, while both *participant* and *text* were entered as random effects. Emotions experienced towards correct-outcome sentences were categorized as *negative* (anxiety, confusion, frustration, boredom), *positive* (curiosity, enjoyment), and *other* (surprise and neutral).

## Supplementary information

Supplementary Online Materials

## Data Availability

The data sets can be found on the Open Science Framework (OSF): https://osf.io/rwta8/?view_only=a40eb0f6228f46ca977fa6778b74b046.

## Code availability

The scripts, written in R, are available on OSF.: https://osf.io/rwta8/?view_only=a40eb0f6228f46ca977fa6778b74b046.
