# Supplementary material for: Investigating epistemic emotions experienced while reading refutation texts through a fine-grained measure of emotion
Source: NPJ Sci Learn. 2025 May 15;10:30. doi: 10.1038/s41539-025-00324-3 (PMC12081847; doi:10.1038/s41539-025-00324-3)
Supplement: Supplementary file 1 — Supplementary Online Materials [file 41539_2025_324_MOESM1_ESM.pdf]

Supplementary Online Materials

Investigating epistemic emotions experienced while reading refutation texts through a fine-grained measure of emotion

Supplement 1. Examining knowledge revision across refutation texts

Supplementary Table 1.

Means, standard deviations, minimums and maximums for metrics of knowledge posttest by text conditions.

Text Conditions	Standard Refutation				Positive Refutation				Negative Refutation				Non-Refutation Control			
	M	*SD*	*Min*	*Max*	*M*	*SD*	*Min*	*Max*	*M*	*SD*	*Min*	*Max*	*M*	*SD*	*Min*	*Max*
postTF	.78	.42	0	1	.73	.44	0	1	.76	.43	0	1	.78	.42	0	1
postExplanation	1.30	.81	0	2	1.14	.89	0	2	1.21	.84	0	2	.88	.78	0	2
postCombine	2.08	1.08	0	3	1.88	1.20	0	3	1.97	1.11	0	3	1.66	1.06	0	3

Supplementary Table 2.

Parameter estimates of the random and fixed effects for knowledge revision by text conditions, age, and educational level.

	postTF (GLMM)				*postExplanation (CLMM)*				*postCombine (CLMM)*			
Random effects	Variance		*SD*		Variance		*SD*		Variance		*SD*	
Participant	1.88		1.37		1.38		1.18		1.37		1.17	
Text	.37		.61		.06		.24		.09		.29	
Fixed effects	*β*	*SE*	*z*	*pr(>|z|)*	*B*	*SE*	*Z*	*pr(>|z|)*	*β*	*SE*	*z*	*pr(>|z|)*
Intercept	1.98	.95	2.09	.04	-	-	-	-	-	-	-	-
PreTF	2.31	.35	6.51	<.001	.91	.25	3.64	<.001	1.31	.24	5.43	<.001
Positive Refutation	-.31	.42	-.75	.45	-.39	.30	-1.32	0.19	-.33	.28	-1.16	.24
Negative Refutation	-.38	.43	-.88	.38	-.35	.30	-1.19	0.24	-.34	.28	-1.22	.22
Non-Refutation	-.28	.44	-.64	.52	-1.40	.30	-4.67	<.001	-1.15	.28	-4.10	<.001
Age	-.03	.02	-1.54	.13	-.04	.02	-2.83	.005	-.04	.02	-2.72	.007
Edu: Technical/community college	-.63	.63	-1.00	.32	.03	.50	.07	.95	-.23	.49	-.47	.64
Edu: Undergraduate	.32	.69	.46	.65	.69	.53	1.29	.20	.52	.52	1.00	.32
Edu: Graduate	.02	1.08	.02	.98	.41	.78	.53	.59	.25	.76	.33	.74
Edu: Doctorate	-.50	1.78	-.28	.78	-.52	1.58	-.33	.74	-.47	1.48	-.32	.75
Model fit	Marginal R^2		Conditional R^2		Marginal R^2		Conditional R^2		Marginal R^2		Conditional R^2	
	.24		.55		.16		.42		.17		.43	

Note. The baseline *text condition* is *Standard Refutation*. The baseline *educational level* is *High School*.

Supplement 2. Emotions experienced in reading refutation texts

Supplementary Table 3.
Means, standard deviations, minimums and maximums for epistemic emotions by text conditions.

Text Conditions	Standard Refutation				Positive Refutation				Negative Refutation				Non-Refutation Control			
	M	*SD*	*Min*	*Max*	*M*	*SD*	*Min*	*Max*	*M*	*SD*	*Min*	*Max*	*M*	*SD*	*Min*	*Max*
Anxiety	.87	1.45	0	7	.85	1.29	0	5	1.31	1.75	0	8	.75	1.27	0	6
Boredom	1.35	2.90	0	13	1.47	3.36	0	21	1.39	3.24	0	20	1.97	3.87	0	19
Confusion	.81	1.19	0	6	.83	1.13	0	5	1.03	1.43	0	7	.67	.97	0	4
Curiosity	2.70	2.31	0	9	3.33	2.64	0	10	2.94	2.82	0	14	2.56	2.08	0	11
Enjoyment	3.49	3.74	0	14	4.46	4.24	0	17	3.62	3.67	0	14	4.42	4.62	0	16
Frustration	.78	1.10	0	4	.87	1.15	0	5	1.59	1.88	0	10	.72	1.16	0	5
Neutral	6.57	4.62	0	19	7.23	5.10	0	20	7.44	5.60	0	23	8.53	6.03	0	22
Surprise	1.20	1.92	0	8	1.63	1.96	0	9	1.64	2.11	0	9	.93	1.61	0	10

Supplementary Table 4.
Parameter estimates of the random and fixed effects for epistemic emotions by text conditions.

	Anxiety				Boredom				Confusion			
Random effects	Variance		*SD*		Variance		*SD*		Variance		*SD*	
Participant	1.41		1.19		4.11		2.03		.91		.95	
Text	.15		.38		.08		.28		.04		.20	
Fixed effects	*β*	*SE*	*z*	*pr(>|z|)*	*B*	*SE*	*Z*	*pr(>|z|)*	*β*	*SE*	*z*	*pr(>|z|)*
Intercept	-.83	.26	-3.19	.001	-1.26	.37	-3.43	<.001	-.59	.20	-2.95	.003
Positive Refutation	.07	.16	.44	.66	.09	.13	.74	.46	-.01	.16	-.08	.93
Negative Refutation	.54	.15	3.69	<.001	-.01	.13	-.04	.97	.23	.15	1.53	.13
Non-Refutation	-.11	.16	-.69	.49	.40	.12	3.40	<.001	-.23	.17	-1.39	.16
Model fit	Marginal R^2		Conditional R^2		Marginal R^2		Conditional R^2		Marginal R^2		Conditional R^2	
	.02		.60		.01		.75		.01		.49	
	Curiosity				Enjoyment				Frustration			
Random effects	Variance		*SD*		Variance		*SD*		Variance		*SD*	
Participant	.58		.76		1.67		1.29		1.09		1.04	
Text	.02		.15		.07		.27		.12		.35	
Fixed effects	*β*	*SE*	*z*	*pr(>|z|)*	*β*	*SE*	*Z*	*pr(>|z|)*	*β*	*SE*	*Z*	*pr(>|z|)*
Intercept	.76	.14	5.44	<.001	.65	.22	2.95	.003	-.68	.24	-2.91	.004

Positive Refutation	.23	.08	2.77	.006	.25	.07	3.36	<.001	.06	.16	.36	.72
Negative Refutation	.07	.09	.84	.40	.08	.08	1.07	.29	.66	.14	4.68	<.001
Non-Refutation	-.05	.09	-.50	.61	.20	.07	2.72	.007	-.18	.17	-1.09	.28
Model fit	Marginal R^2		Conditional R^2		Marginal R^2		Conditional R^2		Marginal R^2		Conditional R^2	
	.01		.63		.01		.82		.04		.58	

	Neutral				Surprise			
Random effects	Variance		*SD*		Variance		*SD*	
Participant	.81		.90		1.14		1.07	
Text	.03		.16		.06		.24	
Fixed effects	*β*	*SE*	*z*	*pr(>|z|)*	*β*	*SE*	*Z*	*pr(>|z|)*
Intercept	1.59	.15	10.65	<.001	-.32	.21	-1.54	.12
Positive Refutation	.09	.06	1.68	.09	.37	.13	2.94	.003
Negative Refutation	.10	.06	1.85	.06	.26	.12	2.13	.03
Non-Refutation	.26	.05	4.77	<.001	-.26	.14	-1.85	.06
Model fit	Marginal R^2		Conditional R^2		Marginal R^2		Conditional R^2	
	.01		.83		.03		.61	

Note. Standard Refutation is the baseline text condition.

Supplementary Table 5.
Counts for activating and deactivating emotions for each paragraph by text conditions.

	Standard Refutation		Positive Refutation		Negative Refutation		Non-Refutation Control	
Paragraph	activate	deactivate	activate	deactivate	activate	deactivate	activate	deactivate
1 Introduction	245 (49%)	253 (51%)	261 (53%)	235 (47%)	261 (51%)	248 (49%)	259 (51%)	250 (49%)
2 Misinformation	186 (78%)	53 (22%)	246 (73%)	93 (27%)	243 (71%)	99 (29%)	156 (57%)	116 (43%)
3 Refutation explanations	262 (76%)	82 (24%)	271 (78%)	76 (22%)	263 (73%)	96 (27%)	260 (45%)	323 (55%)
4 Continuation	166 (45%)	207 (55%)	274 (49%)	290 (51%)	314 (54%)	269 (46%)	195 (51%)	191 (49%)
5 Closing	58 (29%)	142 (71%)	73 (37%)	124 (63%)	71 (36%)	127 (64%)	84 (42%)	117 (58%)

Note. the values shown in () represent the counts of activating (or deactivating) emotions divided by the total count of emotions reported for that condition. For instance, activating emotions reported in paragraph 1 of the standard refutation constitutes 49%. This calculation is derived from the count of activating emotions (n=245) divided by the sum of all emotions (n=245 + 253).

Supplementary Figure 1. Emotions experienced varied across text conditions within different *paragraphs*.
Two line graphs showing the proportion of activating (or deactivating) emotions reported in each paragraph, categorized by text condition.

Activating emotions

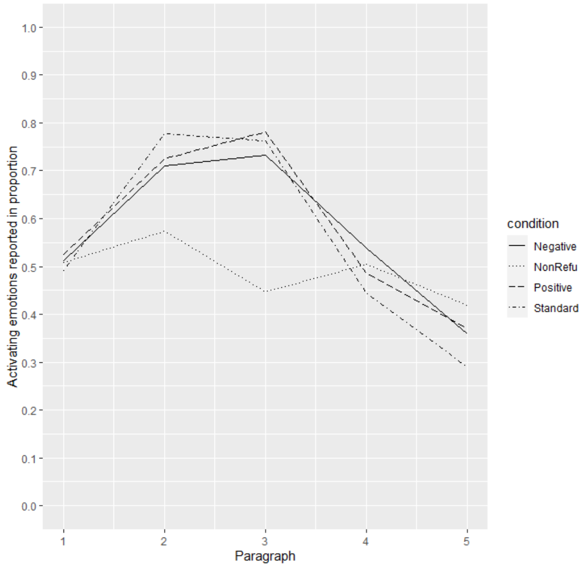

Deactivating emotions

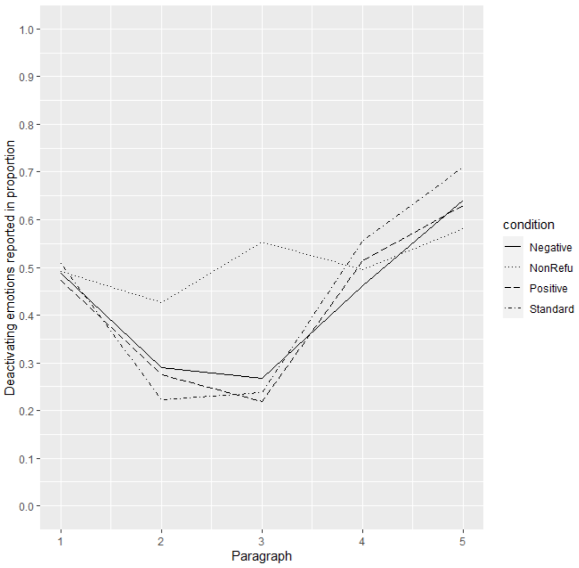

Supplement 3. Effect of in-the-moment reported emotions towards the correct-outcome sentence on knowledge revision

Supplementary Table 6.

Means, standard deviations for knowledge posttest (explanation) as a function of text conditions and emotions towards correct-outcome sentences.

	Standard Refutation		Positive Refutation		Negative Refutation		Non-Refutation Control	
	M	*SD*	*M*	*SD*	*M*	*SD*	*M*	*SD*
Negative emotions	.86	85	.59	.80	.89	.88	.59	.80
Positive emotions	1.55	.74	1.42	.83	1.33	.76	.92	.75
Other	1.39	.70	1.19	.88	1.25	.84	1.00	.72

Supplementary Table 7.

Parameter estimates of the random and fixed effects for knowledge posttest (explanation) by text conditions and emotions towards correct-outcome sentences.

	postExplanation			
Random effects	Variance		*SD*	
Participant	1.53		1.24	
Text	.11		.33	
Fixed effects	*β*	*SE*	*z*	*pr(>|z|)*
Positive Refutation	-.55	.31	-1.77	.08
Negative Refutation	-.40	.31	-1.30	.19
Non-Refutation	-1.43	.31	-4.53	<.001
Correct-outcome sentences: other	.59	.33	1.80	.07
Correct-outcome sentences: positive	.84	.35	2.42	.02
Model fit	Marginal R^2		Conditional R^2	
	.07		.38	

Note. The baseline *text condition* is *Standard Refutation*. The baseline *emotion towards correct-outcome sentences* is *negative emotions.*

Supplementary Table 8.

Parameter estimates of the multiple comparisons of means for text conditions and emotions towards correct-outcome sentences.

postExplanation	*B*	*SE*	*Z*	*pr(>|z|)*
Standard Refutation vs. Positive Refutation	.56	.31	1.77	.46
Standard Refutation vs. Negative Refutation	.40	.31	1.30	1.00
Standard Refutation vs. Non-Refutation	1.43	.32	4.53	<.001
Positive Refutation vs. Negative Refutation	-.15	.31	-.51	1.00
Positive Refutation vs. Non-Refutation	.87	.31	2.84	.03
Negative Refutation vs. Non-Refutation	1.03	.30	3.40	.004
Correct-outcome sentences: Negative vs. Other	-.59	.33	-1.80	.21
Correct-outcome sentences: Negative vs. Positive	-.84	.35	-2.42	<.05
Correct-outcome sentences: Other vs. Positive	-.25	.30	-.83	1.00
